# A Potential Role of Cyclic Dependent Kinase 1 (CDK1) in Late Stage of Retinal Degeneration

**DOI:** 10.3390/cells11142143

**Published:** 2022-07-07

**Authors:** Jiaming Zhou, Per Ekström

**Affiliations:** Ophthalmology, Department of Clinical Sciences Lund, Lund University, 223 62 Lund, Sweden; per.ekstrom@med.lu.se

**Keywords:** cell death, retinal degeneration, CDK1, organotypic retinal explant culture

## Abstract

Cyclin dependent kinase 1 (CDK1) has long been known to drive the cell cycle and to regulate the division and differentiation of cells. Apart from its role in mitosis regulation, it also exerts multiple functions as a protein kinase, including engagement in cell death, possibly via a cell cycle-independent mechanism. The latter is suggested, since CDK1 re-expression can be found in non-dividing and terminally differentiated neurons in several neurodegeneration models. However, the details of if and how CDK1 might be involved in the neurodegenerative condition, retinitis pigmentosa (RP), which displays progressive vision loss, are unclear. In the present study, we investigated CDK1 in degenerating RP photoreceptors of the *rd1* RP model, including whether there is a link between this kinase and the cGMP-PKG system, which is regarded as a disease driver. With experiments performed using either in vivo retinal tissue or in vitro material, via organotypic retinal explants, our results showed that CDK1 appears in the photoreceptors at a late stage of their degeneration, and in such a position, it may be associated with the cGMP-PKG network.

## 1. Introduction

Cyclin dependent kinase 1 (CDK1), also known as cell division control protein 2, has been identified within the main components of the cyclin dependent kinase family to drive the cell cycle in eukaryotic cells. Here, its activation is achieved via the formation of a cyclin B-CDK1 complex, with the subsequent phosphorylation of associated substrates [1]. Beyond being a mitosis regulator, CDK1 functions in transcriptional regulation, cell polarization, and DNA damage repair, and recently, its participation in cell death has also been reported [2]. Interestingly, the re-expression of this cell cycle-related kinase has been found in post-mitotic neurons, which are seen as non-dividing and terminally differentiated cells in the scenario of cell death [3]. These discoveries raise the possibility that CDK1 may be implicated in cell death via a cell cycle-independent mechanism.

Despite studies indicating that CDK1 triggers neuron death in several neurodegenerative conditions, our understanding of this protein in retinitis pigmentosa (RP), a group of inherited degenerative and blinding retinal diseases without any effective treatment, is currently still at a very early stage. The aim of the present study was to use a mouse-based RP model to explore if CDK1 may have a place in degenerating RP photoreceptors, where we focused on the cyclic GMP (cGMP)-protein kinase G (PKG) system, which has been proven to be at least a partial driver of the disease [4]. The downstream pathway of this system thus deserves further investigation to reveal more details of the degeneration mechanism.

## 2. Materials and Methods

Mice, C3H rd1/rd1 (*rd1*; [5]), and control C3H wild-type (wt), were kept and bred in-house under standard white cyclic lighting, with free access to food and water, and used irrespective of sex. Postnatal day (P) 0 was defined as the day of birth of the animal, with the day following this considered as P1, etc.

### 2.1. Organotypic Retinal Explant Culture

Retinal explants were generated from P5 *rd1* retinas, as previously described [6]. Culturing membrane inserts with the explants were put into six-well culture plates, with 1.5 mL serum-free medium in each well. Plates were incubated at 37 °C with a 5% CO_2_ atmosphere, and the medium was replaced every two days. No treatment was added during the culture in the first 2 days. After this, i.e., at a time that equals P7, the cultures were exposed to 50 µM Rp-8-Br-PET-cGMPS (PKG inhibitor; Cat#: P 007, Biolog Life Science Institute, Bremen, Germany) for 4 days, with the endpoint equivalent to P11. The compound was also used at the same concentration for 2 h and 2 days treatment of the explants, such that no treatment was added during the culture until 2 h and 2 days before the endpoint of the protocol. These were complemented with their corresponding untreated controls receiving the same amount of solvent (H_2_O).

### 2.2. Cryosection

Retinal tissues from *rd1* and wt in vivo at P11, as well as cultured explants from *rd1*, were treated with 4% formaldehyde for 2 h, washed for 3 × 15 min in phosphate-buffered saline (PBS), cryoprotected in PBS + 10% sucrose for overnight at 4 °C, and subsequently with PBS + 25% sucrose for 2 h. After embedding, 12 μm thick retinal cross-sections were cut and collected from a HM560 cryotome (Microm, Walldorf, Germany). The sections were stored at −20 °C for later usage. Cryosections were then used for immunostaining.

### 2.3. Immunostaining and TUNEL

For immunostaining, briefly, the cryosections were dried at room temperature for 15 min and rehydrated in PBS. Then, they were blocked with 1% bovine serum albumin (BSA) + 0.25% Triton X100 + 5% goat serum in PBS at room temperature for 45 min. The primary antibody anti-CDK1 (Cat#: MA5-17162, ThermoFisher, Waltham, MA, USA) and anti-acetylated-lysine (Cat#:9441S, Cell Signaling, Danvers, MA, USA) was diluted with 1% BSA and 0.25% Triton X100 in PBS (PTX) and incubated at 4 °C overnight; a no-primary antibody control ran in parallel. Sections were washed 3 × 5 min each in PTX, and incubated with a donkey anti-mouse IgG (H+L) highly cross-adsorbed secondary antibody, Alexa Fluor™ Plus 488 (Cat#A-32766, ThermoFisher), and goat anti-rabbit IgG (H+L) highly cross-adsorbed secondary antibody, Alexa Fluor™ 594 (Cat # A-11037, ThermoFisher, Waltham, MA, USA) at 1:800 dilution in PTX. After 3 × 5-min PBS washes, the sections were mounted with Vectashield DAPI (Vector, Burlingame, CA, USA).

Fluorescent terminal deoxynucleotidyl transferase dUTP nick end-labeling (TUNEL) assay (12156792910, Roche Diagnostics, Mannheim, Germany) was performed according to the manufacturer’s instructions, and used on cryosections to evaluate the cell death.

### 2.4. Microscopy and Image Processing

A Zeiss Imager Z1 Apotome Microscope (Zeiss, Oberkichen, Germany), with a Zeiss Axiocam digital camera was used for microscopy observations. Image generation and contrast enhancement were performed identically for all images via the ZEN2 software (blue edition). The immunostaining was analyzed for staining differences via three sections each, from three to five animals for each condition, after which the fluorescent intensities of positive cells randomly distributed within the area of interest (outer nuclear layer, ONL, i.e., the photoreceptor layer) were assessed. Fluorescence intensity was captured and analyzed using ImageJ software (version 1.53a, NIH, Bethesda, MD, USA). The freehand selection function of the program was used to target the ONL, after which, the fluorescence intensity was calculated with the measure function. The values of all sections from the same animal were averaged.

### 2.5. Statistical Analysis

The values of CDK1-positive photoreceptor percentages, either in tissues or in explants, were analyzed using Student’s *t*-test. Significance was defined as *p* < 0.05.

## 3. Results

To determine whether CDK1 is expressed in photoreceptors (PRs) undergoing degeneration, we immuno-stained for CDK1 in retina sections from the *rd1* RP model and healthy wt mice. The *rd1* is a fast progressive retinal degeneration model, due to a mutation in the gene for phosphodiesterase 6B (PDE6B), that causes the accumulation of the photoreceptor cGMP, and thus the activation of the cGMP-PKG system.

**Figure 1:**
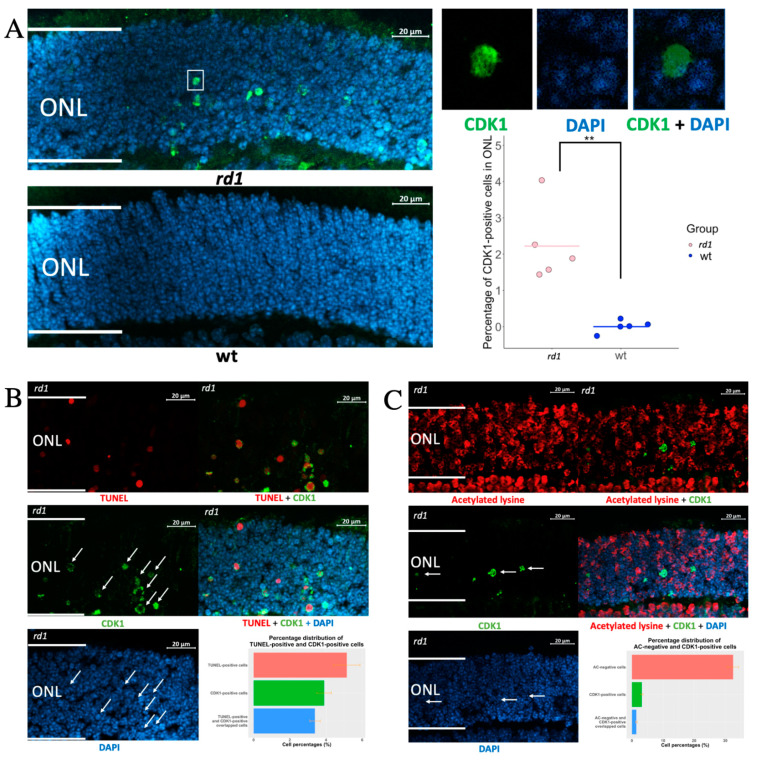
Evaluation of CDK1 expression within the ONL. (**A**) Immunostaining of CDK1 (green) within the ONL of *rd1* and wt strains at P11. The dot chart shows the comparison of CDK1-positive cells within ONL between *rd1* and wt (n = 5); (**B**) Co-staining of CDK1 (green) and TUNEL (red) within ONL from *rd1* retinas, with bar chart showing the distribution of TUNEL-positive and CDK1-positive cells (n = 5). (**C**) Co-staining of CDK1 (green) and acetylated lysine (AC, red) within the ONL from *rd1* retinas, with bar chart showing the distribution of CDK1^+^ cells and AC-negative cells (n = 5). DAPI (blue) was used as nuclear counterstain. For graphs, bars represent mean ± S.D, ** *p* < 0.01. ONL = Outer nuclear layer. Arrows point to the CDK1-positive cells and associated DAPI-positive cells.

At P11, the *rd1* outer nuclear layer (ONL) harbors PRs in various stages, from such that have not yet entered the degeneration process, all the way to cells with increased cGMP, but that are still recognizable as PRs, to TUNEL-positive cells, and such that are only present as fragmented cells due to their degeneration [4]. A quantification of CDK1-positive (CDK^+^) structures showed many such in the *rd1* ONL, while CDK^+^ cells were absent from wt ONL (Figure 1A). Similar to other neurodegenerative diseases, this indicated that CDK1 re-expression also happens in RP. Since the ONL is dominated by photoreceptor nuclei, this in turn suggested that CDK is re-expressed in these structures, which was confirmed by the CDK1 overlap with the nuclear (DNA) counterstain 4,6-diamidino-2-phenylindole (DAPI). However, this overlap was not complete because the CDK1 signal also frequently appeared in the DAPI-negative areas. This indicated that CDK1 was present in some photoreceptor nuclei that had entered a phase of shrinking and condensing, since DAPI is not able to bind to DNA in such situations [7].

To further study the CDK1 relation to photoreceptor death, we co-stained for CDK1 and either acetylated lysine or TUNEL positivity. It has previously been shown that acetylated lysine is absent from RP photoreceptors in a relatively early stage of their degeneration, due to increased histone deacetylase activity [8]. This is in contrast to TUNEL staining, which is well-established as an indicator of cells in a late stage of cell death. The comparison with stainings for acetylated lysine and for TUNEL will therefore be helpful in establishing whether the expression of a defined protein occurs early or late in the degeneration process [4]. Figure 1B (see Supplementary Figure S2 for co-staining of CDK1 and arrestin) shows a very limited overlap between CDK^+^ and acetylated lysine-negative cells, while the CDK^+^ and TUNEL overlap was extensive (Figure 1C; see Supplementary Figure S3 for staining of also P11 wild-type retina). This is compatible with the fact that CDK1 is expressed to only some extent in photoreceptors while they are still in an early stage of degeneration, and that the CDK1 expression increases as they progress to the later stages of degeneration.

**Figure 2:**
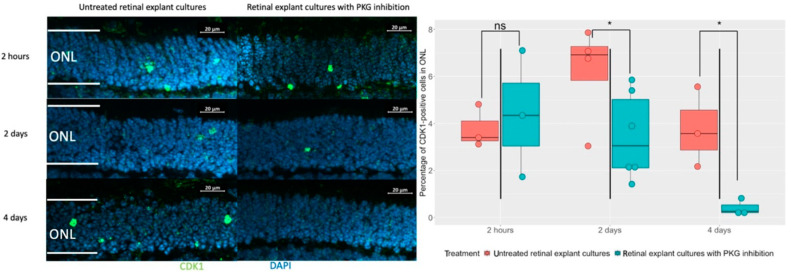
Evaluation of the relation between the cGMP-PKG system and CDK1. Retinal explants from the *rd1* strain were treated with 50 μM Rp-8-Br-PET-cGMPS (PKG inhibitor) for either 2 h, 2 days, or 4 days towards the end of the culturing, and complemented with their untreated counterparts. Left panel shows immunostaining of CDK1 (green) within the ONL in *rd1* retinal explants with different lengths of PKG inhibition, as well as their untreated peers. DAPI (blue) was used as nuclear counterstain. The box chart shows the comparison of CDK1^+^ photoreceptors between retinal explants treated with PKG inhibitor and controls at different lengths of treatment (n = 3–6). * *p* < 0.05. ONL = Outer nuclear layer.

The cGMP-PKG system is suggested as a disease driver of retinal degeneration, not the least in the *rd1* model, where the inhibition of PKG has been repeatedly shown to counteract the degeneration, including in studies based on retinal explants [4,9]. It would hence be informative to assess whether CDK1 is downstream of the cGMP-PKG activities, or whether this represents an independent fatal mechanism. We therefore manipulated the PKG activity via a cGMP analogue with PKG inhibitory actions [10] during organotypic explant culturing between P5 and P11. The latter involved various pharmacological treatment lengths of either the final 2 h, 2 days, or 4 days of the culturing period, all ending at P11, and these were complemented with the retinal explant from the wt as a control (Supplementary Figures S1 and S4). Compared to the non-treated counterparts, we could not find any alteration of CDK1 expression after 2 h of PKG inhibition, while it was evidently less expressed after 2 days and 4 days of treatment (Figure 2). We note that the effect on CDK1 expression after 2 days of PKG inhibition is in contrast to the lack of effect on TUNEL staining from the same treatment [6]. This may be related to the fact that while CDK1 expression according to our overlap analyses is increased late in the degeneration process, it is still early enough to be affected by the PKG inhibition before this reduces TUNEL staining. All in all, this suggested that CDK1 may act as a cGMP-PKG downstream effector, or it is at least related to this system in an indirect way.

## 4. Discussion

A previous study demonstrated the expression of cell cycle proteins, such as cyclin dependent kinase 2 and cyclin dependent kinase 4, in *rd1* [11], but details of CDK1 expression during retinal degeneration have, to our knowledge, not been reported. Here, we show that CDK1 is expressed in degenerating photoreceptors, and mainly in the later stages of cell death, which could mean either that CDK1 takes part in the degeneration process, or that it is involved in a counteractive response. Interestingly, Lv et al. recently showed that reducing CDK1 in retinal degeneration has protective effects, since CDK1 knock-out via genetic modification, as well as CDK1 inhibition via intravitreal injection, resulted in increased photoreceptor survival, albeit not long-lasting [12]. In the light of this, the role of this kinase during RP deserves more investigation to assess its exact function in the cell death process.

In addition, our data of lower CDK1 expression after PKG inhibition suggest an association between this kinase and the cGMP-PKG system, possibly as one of the cGMP-PKG downstream signaling targets. Further research regarding CDK1 phosphorylation under cGMP-PKG regulation is needed to establish whether it constitutes a direct substrate of this system, or is involved in the network via an indirect route. A CDK1 functional study indicated that CDK1 upregulates mitochondrial respiration to drive the cell cycle, whose progression requires a large amount of energy [13]. It is universally accepted that photoreceptors are one of the most energy demanding cells [14], and we recently proposed that PKG inhibition may alleviate retinal degeneration via the positive regulation of oxidative phosphorylation [6]. As a speculation, the progressively decreased CDK1 expression within photoreceptors after 2 and 4 days of treatment could then mean that the oxidative stress of photoreceptors was to some extent alleviated after PKG inhibition, relieving CDK1 from its function to upregulate mitochondrial respiration.

All in all, our study suggests that CDK1 has functions during retinal degeneration, and in particular, in the late stages of photoreceptor death. Future studies may be directed towards the possibility that CDK1 counteracts oxidative stress via the increase of respiration as part of the cGMP-PKG network.

## Data Availability

The data presented in this study are available in Supplementary Figures S1–S4.

## Supplementary Materials

The following supporting information can be downloaded at: https://www.mdpi.com/article/10.3390/cells11142143/s1. Figure S1. Co-staining for CDK1 and TUNEL in a *rd1* retinal explant at a timepoint equivalent to P11. Figure S2. Co-staining for CDK1 and arrestin within ONL from *rd1* model. Figure S3. Evaluation of acetylated lysine (AC, red) expression within ONL from wt and its *rd1* counterpart at P11, and DAPI (blue) was used as a nuclear counterstain. Figure S4. Immunostaining of CDK1 expression (green) within ONL of a P11 retinal explant from the wt strain (as a control to its *rd1* counterpart), and DAPI (blue) was used as a nuclear counterstain.

## References

[1] Li J., Qian W., Sun Q. (2019). Cyclins regulating oocyte meiotic cell cycle progression. Biol. Reprod..

[2] Liao H., Ji F., Ying S. (2017). CDK1: Beyond cell cycle regulation. Aging.

[3] Vincent I., Jicha G., Rosado M., Dickson D. (1997). Aberrant Expression of Mitotic Cdc2/Cyclin B1 Kinase in Degenerating Neurons of Alzheimer’s Disease Brain. J. Neurosci..

[4] Power M., Das S., Schütze K., Marigo V., Ekström P., Paquet-Durand F. (2020). Cellular mechanisms of hereditary photoreceptor degeneration—Focus on cGMP. Prog. Retin. Eye Res..

[5] Sanyal S., Bal A.K. (1973). Comparative light and electron microscopic study of retinal histogenesis in normal and rd mutant mice. Z. Anat. Entwicklungs..

[6] Zhou J., Rasmussen M., Ekström P. (2021). cGMP-PKG dependent transcriptome in normal and degenerating retinas: Novel insights into the retinitis pigmentosa pathology. Exp. Eye Res..

[7] Daniel B., DeCoster M. (2004). Quantification of sPLA2-induced early and late apoptosis changes in neuronal cell cultures using combined TUNEL and DAPI staining. Brain Res. Protoc..

[8] Sancho-Pelluz J., Alavi M., Sahaboglu A., Kustermann S., Farinelli P., Azadi S., van Veen T., Romero F., Paquet-Durand F., Ekström P. (2010). Excessive HDAC activation is critical for neurodegeneration in the *rd1* mouse. Cell Death Dis..

[9] Vighi E., Trifunović D., Veiga-Crespo P., Rentsch A., Hoffmann D., Sahaboglu A., Strasser T., Kulkarni M., Bertolotti E., van den Heuvel A. (2018). Combination of cGMP analogue and drug delivery system provides functional protection in hereditary retinal degeneration. Proc. Natl. Acad. Sci. USA.

[10] Butt E., Pöhler D., Genieser H.G., Huggins J.P., Bucher B. (1995). Inhibition of cyclic GMP-dependent protein kinase-mediated effects by (Rp)-8-bromo-PET-cyclic GMPS. Br. J. Pharmacol..

[11] Zencak D., Schouwey K., Chen D., Ekstrom P., Tanger E., Bremner R., van Lohuizen M., Arsenijevic Y. (2013). Retinal degeneration depends on Bmi1 function and reactivation of cell cycle proteins. Proc. Natl. Acad. Sci. USA.

[12] Lv Z., Xiao L., Tang Y., Chen Y., Chen D. (2021). Rb deficiency induces p21cip1 expression and delays retinal degeneration in *rd1* mice. Exp. Eye Res..

[13] Wang Z., Fan M., Candas D., Zhang T., Qin L., Eldridge A., Wachsmann-Hogiu S., Ahmed K., Chromy B., Nantajit D. (2014). Cyclin B1/Cdk1 Coordinates Mitochondrial Respiration for Cell-Cycle G2/M Progression. Dev. Cell.

[14] Okawa H., Sampath A., Laughlin S., Fain G. (2008). ATP Consumption by Mammalian Rod Photoreceptors in Darkness and in Light. Curr. Biol..

