# Supplementary material for: A Potential Role of Cyclic Dependent Kinase 1 (CDK1) in Late Stage of Retinal Degeneration
Source: Cells. 2022 Jul 7;11(14):2143. doi: 10.3390/cells11142143 (PMC9317054; doi:10.3390/cells11142143)
Supplement: Supplementary file 1 [file cells-11-02143-s001.zip › cells-1728226-supplementary.pdf]

Supplementary Materials

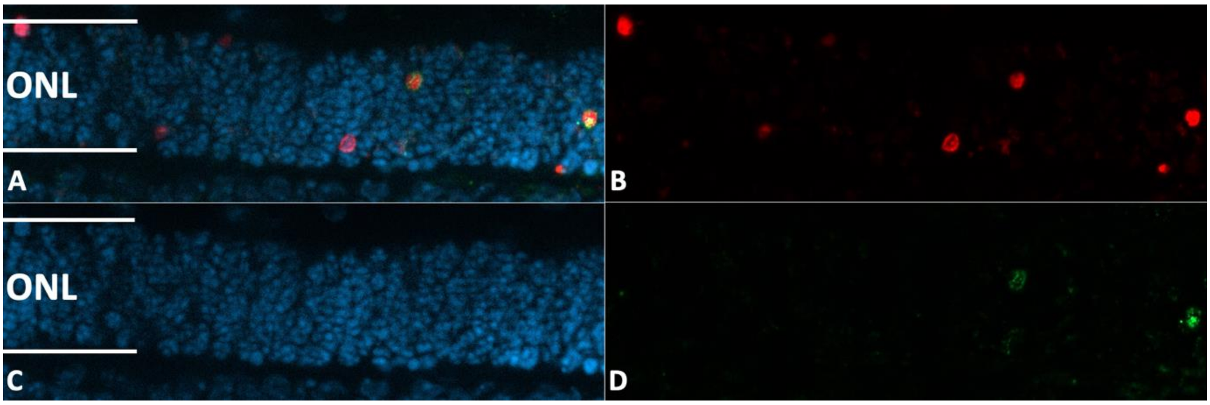

Figure S1. Co-staining for CDK1 and TUNEL in a rd1 retinal explant at a timepoint equivalent to P11. CDK1 was stained in green and TUNEL was stained in red, while DAPI (blue) was used as a nuclear counterstain. A: Immunostaining with multiple channels (red, green and blue). B: Single channel to represent TUNEL-positive cells. C: Single channel to represent DAPI. D: Single channel to represent CDK1-positive cells.

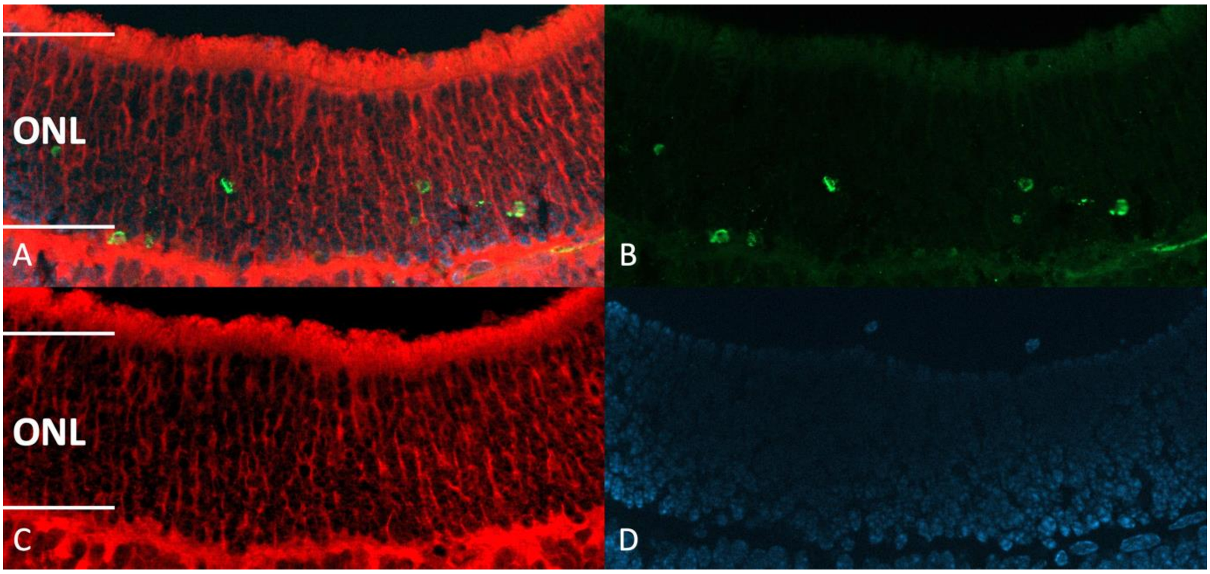

Figure S2. Co-staining for CDK1 and arrestin within ONL from rd1 model. CDK1 was stained in green and arrestin was stained in red, while DAPI (blue) was used as a nuclear counterstain. A: Immunostaining with multiple channels (red, green and blue). B: Single channel to represent CDK1-positive cells. C: Single channel to represent arrestin-positive cells. D: Single channel to represent DAPI.

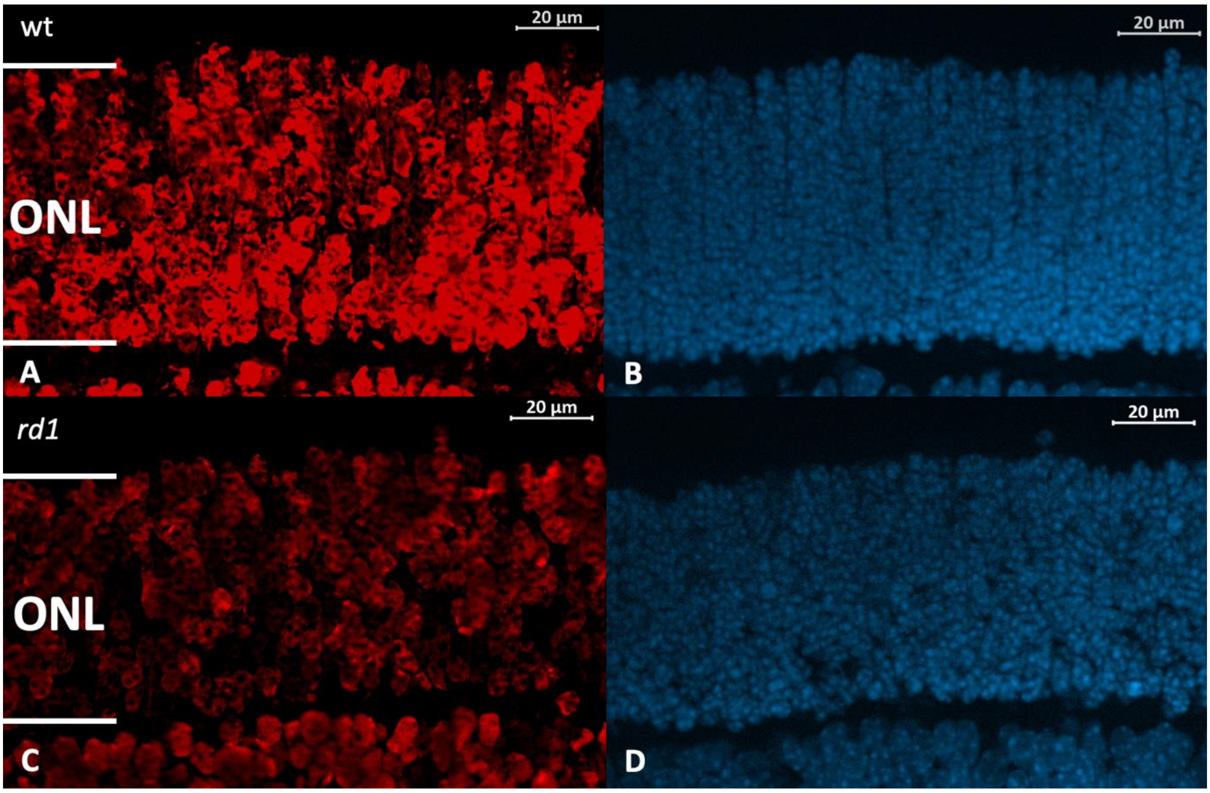

Figure S3. Evaluation of acetylated lysine (AC, red) expression within ONL from wt and its rd1 counterpart at P11, and DAPI (blue) was used as a nuclear counterstain. A: Immunostaining with multiple channels (red and blue). A, B: Single channel to represent AC-positive cells and DAPI, respectively in ONL from in a wt retina. C, D: Single channel to represent AC-positive cells and DAPI, respectively in ONL in an rd1 retina.

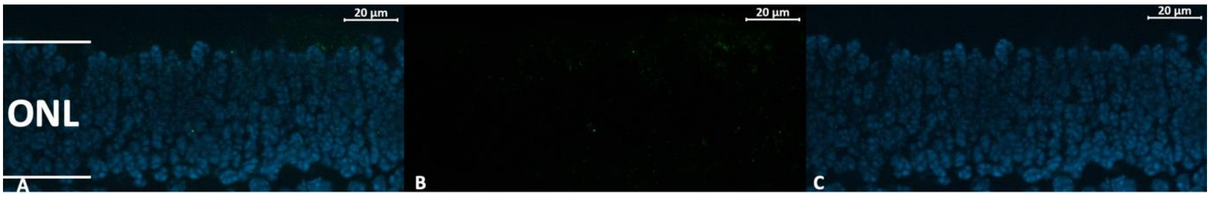

Figure S4. Immunostaining of CDK1 expression (green) within ONL of a P11 retinal explant from the wt strain (as a control to its rd1 counterpart), and DAPI (blue) was used as a nuclear counterstain. A: Immunostaining with multiple channels (green and blue). B: Single channel to represent CDK1-positive cells. C: Single channel to represent DAPI. Note the absence of green CDK1 signal.
